# Migration pelvienne de broche guide dans la chirurgie des fractures de hanche: à propos de 3 cas

**DOI:** 10.11604/pamj.2015.21.112.6541

**Published:** 2015-06-10

**Authors:** Mohamadou Lamine Guèye, Ousmane Thiam, Alpha Oumar Touré, Mamadou Seck, Mamadou Cissé, Ousmane Kâ, Madieng Dieng, Abderahmane Dia, Cheikh Tidiane Touré

**Affiliations:** 1 Service de Chirurgie Générale, Hôpital Aristide Le Dantec, Dakar, Sénégal

**Keywords:** Migration pelvienne, minilaparotomie, broche-guide, hanche, ostéosynthèse, fracture, complication, pelvic migration, minilaparotomy, guide pin, hip, osteosynthesis, fracture, complication

## Abstract

La migration de matériel d'ostéosynthèse est une complication bien connue du traitement chirurgical des fractures. Toutefois, la migration pelvienne de broche guide dans la chirurgie des fractures de la hanche est un incident rare et potentiellement grave. Nous rapportons 3 observations dans lesquelles on notait une migration de broche guide dans le pelvis lors d'une ostéosynthèse dela hanche de type DHS. L'indication chirurgicale était une fracture per-trochantérienne dans 2 cas et une fracture du col fémoral type 4 de Garden dans 1 cas. Une minilaparotomie permettait d'objectiver une plaie du ligament large gauche et un hématome ligamentaire dans 1 cas, tandis que dans les 2 autres cas, il n'y avait pas de lésion viscérale. L'extraction de la broche a été effectuée dans les 3 cas. Les suites opératoires ont été simples chez tous les patients.

## Introduction

La migration de matériel d'ostéosynthèse est une complication fréquente dans le traitement chirurgical des fractures [1, 2]. Cependant, la migration pelvienne peropératoire de broche guide dans la chirurgie des fractures de hanche est une entité rare [2]. Il s'agit d'un incident grave pouvant engager le pronostic vital du patient, en raison de la richesse du pelvis en viscères et gros vaisseaux. Nous rapportons notre expérience dans la prise en charge de 3 cas de migration pelvienne peropératoire de broche guide survenue au cours d'une ostéosynthèse pour fracture de hanche. Les aspects diagnostiques, lésionnels, thérapeutiques et les suites opératoires ont été étudiés.

## Patient et observations

**Figure 1:**
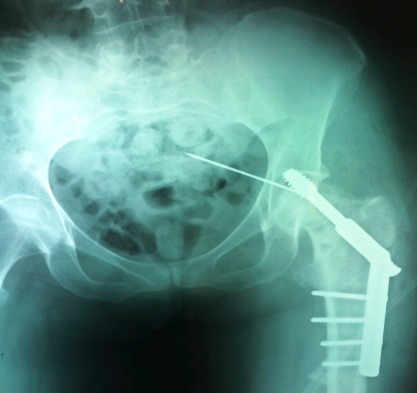
Radiographie du bassin montrant la broche intra-pelvienne (observation 1)

**Figure 2:**
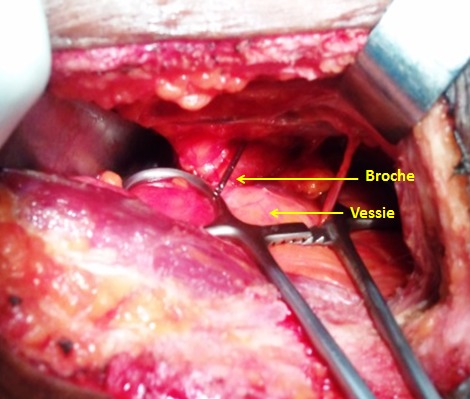
Vue per-opératoire de la broche en regard de la vessie (observation 1)

**Observation 1:** un patient de 32 ans était admis au bloc opératoire pour une ostéosynthèse de la hanchepar vis DHS, indiquée pour une fracture per-trochantérienne avec un arrachement du petit trochanter. Après un abord par la voie de Watson Jones, une broche guide était mise en place pour un taraudage. Lors de ce taraudage, sont survenues une cassure et une migration de la broche guide dans le pelvis. Ceciétait confirmé par l'amplificateur de brillance (Figure 1). Un abord sous-péritonéal par une incision médiane sous-ombilicale permettait de retrouver la broche guide en regard de la vessie et son extraction par une pince gouge (Figure 2). Il n'y avait pas de lésion viscérale. Les suites opératoires étaient simples.

**Observation 2:** une patiente de 49 ans était admise pour un traumatisme fermé de lahanche survenu au décours d'une chute de sa hauteur. A la radiographie du bassin, on objectivait une fracture du col fémoral type 4 de Garden. Une réduction et une ostéosynthèse de type DHS étaient alors indiquées. Un incident opératoire à type de cassure et de migration de la broche guide étaitsurvenu lors du taraudage. Une radiographie du bassin objectivait une protrusion pelvienne de la broche guide. La tentative d'extraction par voie trans-acétabulaire s'est soldée par un échec. Une minilaparotomie médiane permettait de retrouver une broche de 7 cm en regard de la veine iliaque externe gauche. Il n'y avait pas de plaie viscérale. L'extraction de la broche a été faite par une pince de Péan. Les suites opératoires étaient sans particularités.

**Figure 3:**
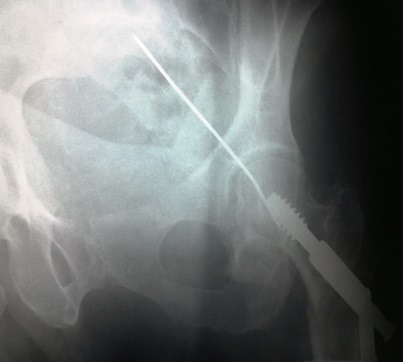
Radiographie du bassin montrant la broche en situation intra-pelvienne (observation 3)

**Figure 4:**
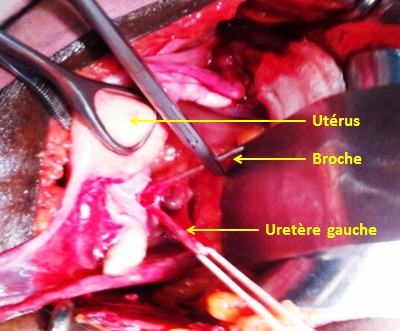
Vue per-opératoire de la broche transfixiant le ligament large gauche (observation 3)

**Observation 3:** une patiente de 82 ans était admise pour une fracture per-trochantérienne avec un arrachement du petit trochanter à la suite d'une chute de sa hauteur. Une réduction et une ostéosynthèse de la hanche par vis DHS était alors indiquées. Après un abord par la voie de Watson Jones, un taraudage était réalisé avec une broche guide occasionnant la migration de la broche dans le pelvis. Une radiographie du bassin objectivait la protrusion pelvienne de la broche (Figure 3). Une tentative d'extraction de la broche par traction sur son bout externe s'est soldée par un échec. Un abord par voie de Pfannenstiel permettait de retrouver la broche qui transfixiait le ligament large gauche avec un hématome intraligamentaire (Figure 4). L’élargissement de la plaie ligamentaire permettait de visualiser l'uretère gauche qui était indemne. Il était réalisé une extraction de la broche et une fermeture de la brèche ligamentaire. Les suites opératoires étaient simples.

## Discussion

L'usage de broche-guide est fréquent dans la chirurgie des fractures de hanche. Elles sont utilisées pour forer le trajet de la vis DHS [2]. La migration pelvienne de ces broches est un incident rare, qui peut menacer le pronostic vital du patient à court et à moyen termes [2]. Dans notre série, il s'agissait d'une cassure et d'une migration pelvienne de la broche survenues lors du taraudage. L'incident était confirmé en per-opératoire par l'amplificateur de brillance dans 1 cas, et par une radiographie du bassin réalisée sur table opératoire dans les 2 autres cas. Des cas de migration de broche diagnostiqués en post-opératoire ont également été rapportés dans la littérature [3]. Plusieurs facteurs peuvent expliquer cet incident. En effet, l'usage d'un taraud mal nettoyé provoquerait une augmentation de la température et une nécrose tissulaire locale. L'accumulation de débris nécrotiques augmenterait la résistance à la pénétration de la broche guide dans l'os [4, 5]. Ainsi, la force imprimée à la broche devient plus importante et est à l'origine de sa déformation et de sa cassure. En outre, l'usage répété des broches favorise la survenue de cet incident [2]. Le chirurgien devrait s'assurer que les broches mises à sa disposition sont résistantes et que leur bout est aiguisé [3]. C'est ainsi que dans leur étude, Ashford et al recommandaient de plier systématiquement les broches utilisées pour éviter leur usage répété [6]. L'extraction de la broche peut se faire par laparotomie ou par voie extrapéritonéale. C'est ainsi que l'extraction par voie ilio-inguinale a été réalisée avec succès dans certains cas [2]. De rares cas d'extraction par voie laparoscopique ont été rapportés [1]. Il s'agissait de migration complète de la broche; ce qui n’était pas le cas de nos patients. Nous avons effectué une laparotomie,vu la profondeur de la protrusion de la broche chez 2 de nos patients. Tandis qu'un abord extrapéritonéal était réalisé chez 1patient. En outre, nous avons noté une plaie du ligament large gauche avec un hématome intraligamentaire dans 1 cas. Dans les 2 autres cas on ne notait pas de lésion viscérale. Des cas de plaies rectale, vésicale, intestinale et vasculaire secondaires à la migration de broche-guide ont également été rapportés [1]. La gravité de cet incident est en rapport avec la richesse du pelvis en viscères et gros vaisseaux. Sa prévention passe par l'usage de broches et de matériel adaptés; et par le contrôle radiographique systématique en post opératoire immédiat

## Conclusion

La migration pelvienne de broche guide est un incident grave qui peut menacer le pronostic vital du patient. Sa prise en charge doit être rapide et adaptée au bilan lésionnel. Sa prévention passe par l'usage d'un matériel adapté et par une bonne technique opératoire. La collaboration pluridisciplinaire est indispensable pour la bonne prise en charge de cet incident.
